# Immunoglobulins in COVID-19 pneumonia: from the acute phase to the recovery phase

**DOI:** 10.1186/s40001-024-01824-5

**Published:** 2024-04-06

**Authors:** Joaquim Peraire, Graciano García-Pardo, Silvia Chafino, Alba Sánchez, Maryluz Botero-Gallego, Montserrat Olona, Sonia Espineira, Laia Reverté, Vasso Skouridou, Óscar M. Peiró, Fréderic Gómez-Bertomeu, Francesc Vidal, Ciara K. O’ Sullivan, Anna Rull

**Affiliations:** 1 Hospital Universitari de Tarragona Joan XXIII (HJ23), Tarragona, Spain; 2 Institut Investigació Sanitària Pere Virgili (IISPV), Tarragona, Spain; 3 Centro de Investigación Biomédica en Red de Enfermedades Infecciosas (CIBERINFEC), Instituto de Salud Carlos III, Madrid, Spain; 4 Universitat Rovira I Virgili (URV), Tarragona, Spain; 5 INTERFIBIO Consolidated Research Group, Tarragona, Spain

**Keywords:** SARS-CoV-2, COVID-19 pneumonia, IgA, IgG, IgM, Immunoglobulins

## Abstract

**Background:**

COVID-19 pneumonia causes hyperinflammatory response that culminates in acute respiratory syndrome (ARDS) related to increased multiorgan dysfunction and mortality risk. Antiviral-neutralizing immunoglobulins production reflect the host humoral status and illness severity, and thus, immunoglobulin (Ig) circulating levels could be evidence of COVID-19 prognosis.

**Methods:**

The relationship among circulating immunoglobulins (IgA, IgG, IgM) and COVID-19 pneumonia was evaluated using clinical information and blood samples in a COVID-19 cohort composed by 320 individuals recruited during the acute phase and followed up to 4 to 8 weeks (n = 252) from the Spanish first to fourth waves.

**Results:**

COVID-19 pneumonia development depended on baseline Ig concentrations. Circulating IgA levels together with clinical features at acute phase was highly associated with COVID-19 pneumonia development. IgM was positively correlated with obesity (ρb = 0.156, P = 0.020), dyslipemia (ρb = 0.140, P = 0.029), COPD (ρb = 0.133, P = 0.037), cancer (ρb = 0.173, P = 0.007) and hypertension (ρb = 0.148, P = 0.020). Ig concentrations at recovery phase were related to COVID-19 treatments.

**Conclusions:**

Our results provide valuable information on the dynamics of immunoglobulins upon SARS-CoV-2 infection or other similar viruses.

**Supplementary Information:**

The online version contains supplementary material available at 10.1186/s40001-024-01824-5.

## Background

The coronavirus disease 2019 (COVID-19) pandemic caused by severe acute respiratory syndrome coronavirus-2 (SARS-CoV-2) is a concerning global health issue [1]. SARS-CoV-2 infection can lead to a wide spectrum of clinical manifestations, ranging from asymptomatic to mild, moderate, and ultimately life-threatening outcomes. During the first wave of the COVID-19 pandemic, most patients experienced mild symptoms of upper respiratory tract infection, but approximately twenty percent rapidly progressed to severe illness accompanied by pneumonia. Pneumonia is an acute respiratory infection that causes inflammation in the lungs and represent one of the most repeated clinical features in critically ill patients [2–4]. Of note, around fifty-five percent of these patients died of severe COVID-19 pneumonia, overcoming the mortality rate of mild patients by ten times [5]. The hyperinflammatory response caused by pneumonia could potentially result in acute immune-mediated lung injury, culminating in acute respiratory syndrome (ARDS), hypercoagulation, multiorgan dysfunction, and increased mortality risk [6, 7]. At this stage, the role of humoral immunity in mitigating viral infection through the production of antiviral-neutralizing antibodies is crucial. Accordingly, immunoglobulin (Ig) production in response to SARS-CoV-2 infection can be regarded as a reflex of the host’s humoral status and illness severity [8, 9]. Multiple studies have focused their research on the use of serological Ig levels as biomarkers of immunological protection related to SARS-CoV2 infection [10–16]; although the association between Igs levels and Covid-19 pneumonia is poorly understood. Understanding the factors that prevent the host from building a robust immune response against the infection is essential for an in-depth comprehension of the humoral response's role in the disease’s pathogenesis and progression toward severe illnesses. Hence, to further explore the relationship between humoral immunity and COVID-19 pneumonia development, circulating Ig concentrations were measured in a prospectively longitudinal Spanish COVID-19 cohort (samples at both the acute phase of the infection and at 4 to 8 weeks after the onset of the symptoms) from first to third waves (unvaccinated patients).

## Patients and methods

### Study participants and data collection

**Fig. 1:**
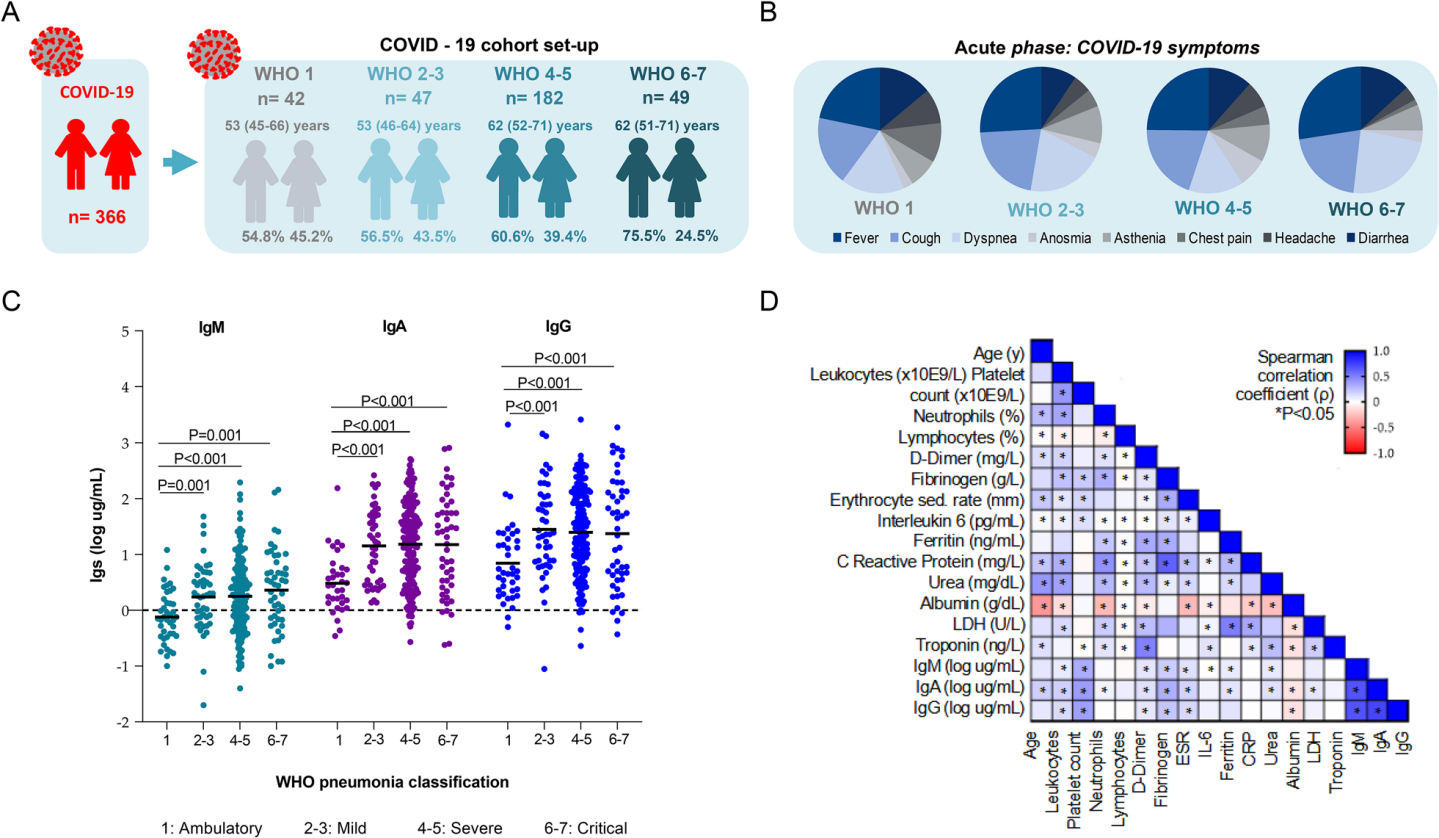
Study design and immunoglobulin concentrations in COVID-19 patients based on Berlin WHO classification criteria during acute phase. **A** The study cohort comprised 366 patients (time of admission). The patients were classified based on Berlin WHO Criteria. **B** Demographic and symptomatology data were recorded at the time of admission. In every group, number of patients (n), sex proportion (in percentage; %) and age range (median and 25th–75th interquartile range) are indicated in the top-right panel. **C** Circulating levels of IgM, IgA and IgG in ambulatory, mild, severe and critical COVID-19 patients according to the Berlin WHO pneumonia classification criteria measured by ELISA are represented by scatter dot plot with mean (wide line). Statistical differences among groups were determined by the nonparametric Kruskal–Wallis test followed by Mann–Whitney U-test. **D** Heatmap showing the Spearman (ρ) correlation coefficient of pairwise comparison analyses between IgM, IgA and IgG concentrations with age and selected inflammatory parameters previously related to COVID-19 disease. The correlation matrix is colour-coded according to the Spearman (ρ) correlation coefficient (− 1:1, red: blue through white), and correlations with statistical significance are indicated with an asterisk as *P < 0.05

The overall COVID-19 cohort comprises 366 patients with SARS-CoV-2 infection confirmed by RT-qPCR, who were recruited between March 2020 and February 2021 (from the first to the third waves) at the Hospital Universitari Joan XXIII, Tarragona. The study did not identify the specific SARS-CoV-2 variants of concern (VOC), but it has been reported that Alpha (lineage 74 B.1.1.7), Beta (lineage B.1.351) and Delta (lineage B.1.617.2) were the predominant circulating variants during this period [17–20]. The longitudinal cohort study included 252 of these patients who were followed up 4 to 8 weeks after illness onset. The participants were grouped by pneumonia severity based on the WHO classification criteria [21] into ambulatory (n = 42) (mild illness without pneumonia; WHO = 1), mild (n = 47) (mild illness presenting pneumonia without hospitalization (WHO 2; n = 46) or with hospitalization but not requiring oxygen (WHO3; n = 1)), severe (n = 182) (hospitalized patients presenting moderate pneumonia who required low-flow oxygen (WHO 4; n = 163) and/or noninvasive ventilation (WHO 5; n = 19)) and critical (n = 49) (patients hospitalized at ICU with severe pneumonia and requiring mechanical ventilation/intubation (WHO 6; n = 22), vasopressors or dialysis (WHO 7; n = 27)). Patients who deceased before reaching the 4 to 8 weeks of follow-up were excluded from the study (n = 46) (WHO 8) (Fig. 1A). Thus, the cohort for the present study comprises 320 individuals (WHO 1 to WHO 7) (Fig. 1A). None of the patients enrolled in the present study had received the SARS-CoV-2 vaccine at the time of blood sampling. The sample size was based on the availability of the samples. Investigators were blinded to disease severity during the analysis. Whereas the analysis was cross-sectional, the patient outcomes were recorded prospectively after inclusion. Oxygen therapies (low- and high-flow oxygen, and non-invasive mechanical ventilation) and intensive care (mechanical ventilation, intubation, vasopressors, or dialysis) are required at admission and/or due to the infection were recorded when applicable. The development of pneumonia was diagnosed by pulmonary radiographic imaging. Symptoms displayed, medicines taken before SARS-CoV-2 infection, pre-existing comorbidities, medicines received for COVID-19 treatment and complications such as pulmonary thromboembolism (PTE) arising from the most acute infections were also considered relevant for subsequent analysis.

### Sample recruitment

The sampling protocol performed included clinical evaluation, blood cell count, and standard biochemical parameters at both time points. Serum samples were stored at -80 ºC at the BioBank-Institut d’Investigació Sanitària Pere Virgili (IISPV) facilities until needed.

### Serum IgA, IgG, and IgM quantification

Serum samples collected in the acute and healing phases of the infection were analyzed for the presence of immunoglobulin G (IgG), immunoglobulin M (IgM), and immunoglobulin A (IgA) to SARS-CoV-2 using an enzyme-linked immunosorbent assay (ELISA) developed in-house as reported previously [22]. Specifically, recombinant SARS-CoV-2 nucleoprotein (50 μL of 5 μg/mL NP in 50 mM carbonate-bicarbonate buffer pH 9.4) was coated overnight at 4 ºC on 96-well immunoplates (Nunc MaxiSorp™, Fisher Scientific). The wells were washed three times with PBS (10 mM phosphate, 137 mM NaCl, 2.7 mM KCl, pH 7.4) containing 0.05% (v/v) Tween-20 (PBST) and then blocked for 30 min with 200 μL of 5% (w/v) skim milk prepared in the same washing buffer. After washing three times, the serum samples (50 μL, diluted 1/100 with PBS) were added to the wells and incubated for 1 h, followed by another three washes. Antigen-bound antibodies were detected using specific antibody-enzyme conjugates (50 μL of goat anti-human IgA-HRP, goat anti-human IgM-HRP or goat anti-human IgG-HRP, diluted 1/20,000 with PBST) after 30 min of incubation. The wells were thoroughly washed five times and the TMB enzyme substrate (50 μL) was finally added. The same volume of 1 M sulfuric acid was added to stop the reaction after 5 min (IgA and IgM) or 7 min (IgG measurements) and the absorbance at 450 nm was recorded. Standard antibody calibration curves were performed in parallel using standards of human IgA, IgM or IgM for coating the wells (ranges of 0.004–16 μg/mL for IgA and IgG and 0.002–8 μg/mL for IgM). A sigmoidal 4-parameter logistical model (GraphPad Prism) was used for the construction of the calibration curves for each antibody. The levels of the antibodies in the serum samples were interpolated from the corresponding curve using the corrected absorbance values (absorbance from antigen-coated wells minus the absorbance from the control wells without antigen). All samples were analysed in triplicate, whereas duplicate measurements were performed for the control wells without viral antigen (to eliminate non-specific signals from serum components) and for the antibody standards used for the calibration curves. Incubation steps were performed at 22 ºC (except for antigen coating) under gentle agitation while 200 μL of PBST was used for each washing step.

### Statistical analysis

Before the statistical analyses, the normal distribution and homogeneity of the variances were tested using a Kolmogorov–Smirnov test. Normally distributed data were expressed as the mean ± standard deviation (SD) or standard error of the mean (SEM) as properly indicated, whereas variables with a skewed distribution were represented as the median (Interquantil range: 25th percentile–75th percentile) or transformed into a decimal logarithm. Statistical differences between groups were performed using the non-parametric Kruskal–Wallis test, Mann–Whitney U-test and the Wilcoxon t test for paired samples. Associations between quantitative variables were evaluated using the Spearman correlation test and correlations between qualitative variables, comorbidities and COVID-19 treatment were calculated using the point-biserial correlation coefficient. Logistic regression analyses and receiver operating characteristic (ROC) curves were employed (stepwise forward selection procedures) to evaluate the potential accuracy of Igs levels with selected parameters for the diagnosis of COVID-19 pneumonia development. Statistical analyses were performed using SPSS (version 21.0, SPSS Inc., Chicago, IL), and graphical representations were generated with GraphPad Prism (version 5.0, GraphPad Inc., San Diego, CA) and PowerPoint software (version 2007). The results were considered significant at P values < 0.05.

## Results

### Baseline Ig concentrations were related to COVID-19 severity

Severe (n = 182, WHO 4–5) and critical (n = 49, WHO 6–7) patients were older (62 years old in both groups) and showed a significant predominance of males (61% and 75%, respectively) as compared to mild (n = 47, WHO 2–3) and ambulatory patients (n = 42, WHO 1; patients without pneumonia) who accounted around 55% of males and had an average age of 53 years (P < 0.001) (Fig. 1A). The most frequent symptoms in this cohort were fever, cough, dyspnea and diarrhea, with a prevalence of 29% in severe patients and 36.7% in critical patients (Fig. 1B). Serum biochemical composition revealed significant lymphopenia (P < 0.001) and monocytopenia (P = 0.007) in both severe and critical patients, but only critical patients showed a significant increase in leukocyte and neutrophil concentrations (P = 0.003 and P < 0.001, respectively) (Additional file 2: Table S1). Circulating D-dimer and troponin were higher in critical patients (P < 0.001), and inflammatory marker concentrations such as fibrinogen, ferritin, and C reactive protein (CRP) were significantly increased with disease severity (P < 0.001). (Additional file 2: Table S1).

IgM, IgA, and IgG concentrations were significantly higher in patients presenting COVID-19 pneumonia (mild, severe, and critical) than in the ambulatory group (P ≤ 0.001). No differences were found among the mild, severe, and critical groups (Fig. 1C). Then, Ig concentrations were correlated with demographic and clinical parameters to better understand the different Ig responses in the acute phase of SARS-CoV-2 infection (Fig. 1D). IgM, IgA and IgG concentrations were positively related to leukocytes (ρ = 0.161, P = 0.003 for IgM, ρ = 0.226, P < 0.001 for IgA and ρ = 0.221, P =  < 0.001 for IgG), platelets (ρ = 0.356, P < 0.001 for IgM, ρ = 0.408, P < 0.001 for IgA and ρ = 0.435, P < 0.001 for IgG), D-dimer (ρ = 0.123, P = 0.025 for IgM, ρ = 0.153, P = 0.006 for IgA and ρ = 0.132, P = 0.017 for IgG), fibrinogen (ρ = 0.230, P < 0.001 for IgM, ρ = 0.324, P < 0.001 for IgA and ρ = 0.256, P < 0.001 for IgG) and erythrocyte sedimentation rate (ESR) (ρ = 0.173, P = 0.004 for IgM, ρ = 0.218, P < 0.001 for IgA and ρ = 0.258, P < 0.001 for IgG). Both IgM and IgA were positively correlated to ferritin (ρ = 0.124, P = 0.038; ρ = 0.139, P = 0.022, respectively) and urea (ρ = 0.108, P = 0.044; ρ = 0.126, P = 0.021, respectively) concentrations. LDH was positively correlated with IgA levels (ρ = 0.131, P = 0.018). Albumin concentrations were negatively correlated with both IgA and IgG concentrations (ρ = − 0.146, P = 0.016 for IgA and ρ = − 0.144, P = 0.016 for IgG). IL-6 was negatively correlated with IgM (ρ = − 0.147, P = 0.014) (Fig. 1D).

### COVID-19 pneumonia development depended on baseline Ig concentrations

**Fig. 2:**
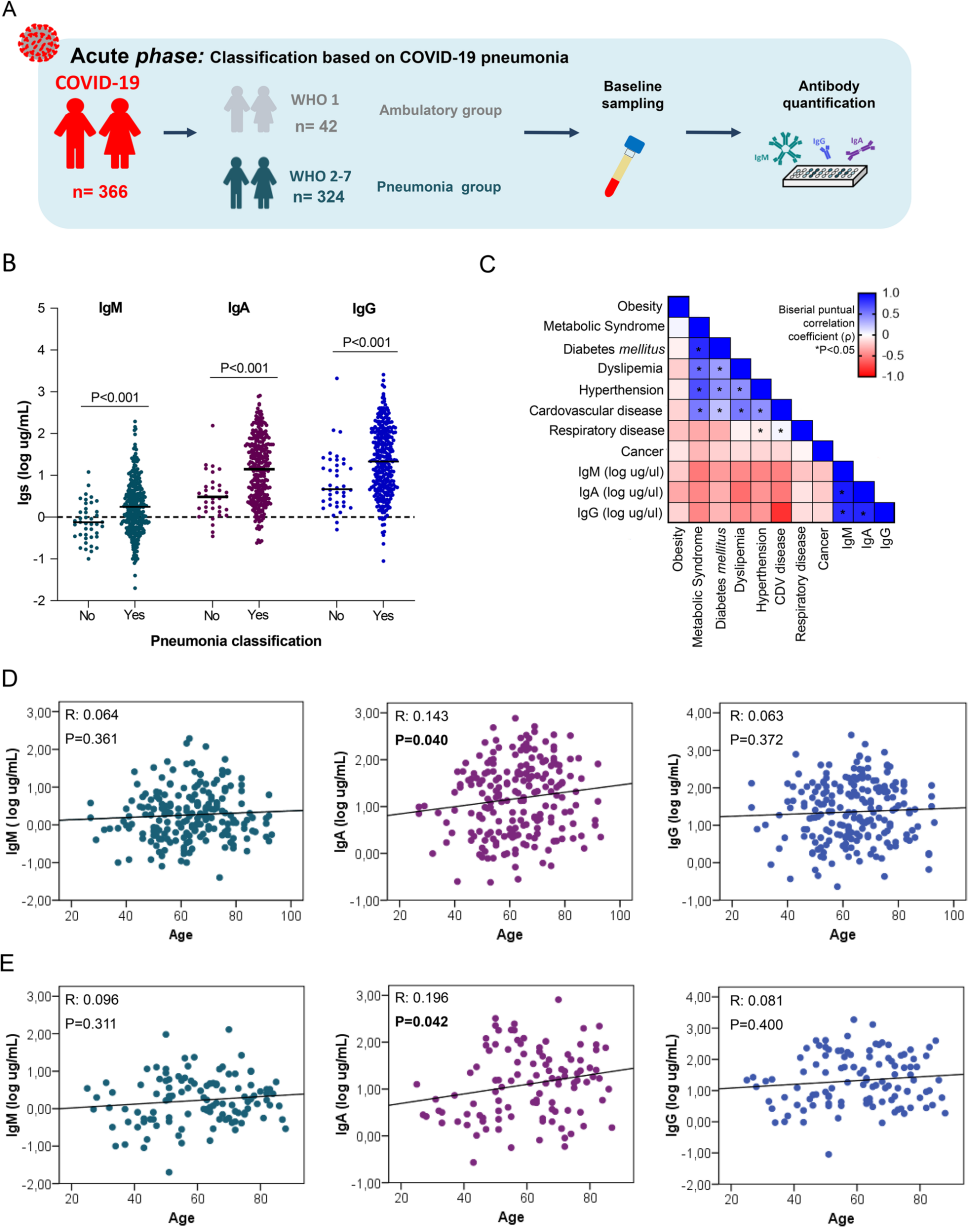
Circulating immunoglobulin concentrations in COVID-19 pneumonia at the time of admission. **A** The study cohort comprised 366 patients were classified based on the absence (WHO 1; Ambulatory group) or presence of pneumonia (WHO 2–7; Pneumonia group). Blood sampling for ELISA analysis was performed at the time of admission. **B** Circulating levels of IgM, IgA and IgG in the no pneumonia group (WHO 1; patients without pneumonia, ambulatory group) and COVID-19 pneumonia group (WHO 2–7) measured by ELISA are represented by scatter dot plot with mean (wide line). Statistical differences among groups were determined by the nonparametric Mann–Whitney U-test. **C** Heatmap showing the point-biserial correlation coefficient (pb) of pairwise comparison analyses between previous comorbidities with levels of the three Igs. The correlation matrix is colour-coded according to the point-biserial correlation coefficient (− 1:1, blue:red through white), and correlations with statistical significance are indicated with an asterisk as *P < 0.05. (D) Individual correlations of each Ig with age in men and (E) women who developed pneumonia (WHO 2–7). Statistical significance are indicated in bold as *P < 0.05

Our results (Fig. 1C) indicated that baseline circulating Ig concentrations were related to the presence of COVID-19 pneumonia independent of the COVID-19 severity degree; thus, patients presenting pneumonia (mild, moderate, severe, and critical) were grouped into a unique group (WHO 2–7, hereinafter named the COVID-19 pneumonia group) and compared to the ambulatory group (WHO 1, no pneumonia) (Fig. 2A). Serum IgM, IgA, and IgG concentrations were significantly increased in the COVID-19 pneumonia group compared to the ambulatory group (P < 0.001) (Fig. 2B).

Clinical and biochemical parameters were analysed based on the new classification criteria (Table 1). The COVID-19 pneumonia group presented a significant increase in fibrinogen (P < 0.001), neutrophils (P = 0.014), C reactive protein (CRP) (P < 0.001), LDH (P < 0.001) and troponin (P = 0.01) concentrations and activated thromboplastin time parameter (P = 0.037), compared to the ambulatory group. Ferritin was significantly higher in the COVID-19 pneumonia group (P = 0.002) with an increase of 165% with respect to the ambulatory group (281 ng/mL versus 747 ng/mL) (Table 1). The ambulatory group presented a significant increase in monocyte concentrations compared to the COVID-19 pneumonia group (P < 0.001). COVID-19 pneumonia was independent of the presence of previous co-morbidities. Oxygen requirement, Azithromycin, corticosteroids and Remdesivir were the most used treatments in the pneumonia group (p < 0.01) (Table 2).

**Table 1:** Baseline demographic, symptomatology and biochemistry characteristics of the COVID-19 study cohort classified by pneumonia

	No Pneumonia^a^ (WHO 1) (n = 42)	Pneumonia^b^ (WHO 2–7) (n = 278)	P-value^c^
Male	23 (54.8)	210 (64.8)	0.135
Age, years	53 (45–66)	63 (52–73)	**0.007**
Oxygen Saturation on Admission (%)	98 (96.7–99)	93 (91–95)	**< 0.001**
**COVID-19 Symptoms**			
Fever	17 (40.5)	242 (74.7)	**< 0.001**
Cough	14 (33.3)	198 (61.1)	**0.001**
Dyspnea	13 (31)	193 (59.5)	**< 0.001**
Anosmia	2 (4.8)	40 (12.3)	0.112
Asthenia	6 (14.3)	85 (26.2)	0.064
Chest Pain	8 (19)	37 (11.4)	0.121
Headache	7 (16.7)	49 (15.1)	0.465
Diarrhea	11 (26.2)	91 (28)	0.486
**Haematological parameters**			
Leukocytes (×10E9/L)	6.2 (4.8–8.1)	6.4 (4.7–8.6)	0.913
Red blood cell count (×10E9/L)	4.3 (4.8–8.1)	4.5 (4.7–8.6)	0.340
Hemoglobin (g/dL)	12.8 (10.4–14.7)	13 (11.8–14)	0.879
Hematocrit (%)	38.3 (31.8–44.8)	39.8 (36.6–42.7)	0.367
Platelet count (×10E9/L)	206 (172.5–235)	219 (168–286)	0.074
Neutrophils (%)	68.8 (58.7–79.7)	76.3 (65.2–83.7)	**0.014**
Lymphocytes (%)	21.5 (12–28.8)	16.9 (10.4–24.6)	0.064
Total lymphocytes (%)	1215 (755–1592.5)	965 (690–1420)	0.158
Monocytes (%)	8 (5.8–10.4)	6.5 (4.6–8.6)	** < 0.001**
**Coagulation parameters**			
Activated thromboplastin time (seg)	29.5 (26.5–32.1)	30.7 (28.8–33)	**0.037**
Prothrombin time (seg)	12.9 (11.7–13.4)	12.7 (12.1–13.5)	0.762
d-Dimer (mg/L)	501.5 (316.2–1191.5)	665.5 (424–1014.5)	0.303
Fibrinogen (g/L)	561 (435–761)	769 (658.5–864)	** < 0.001**
**Inflammatory markers**			
Erythrocyte sedimentation rate (mm)	89 (14–117.5)	55.5 (32–83.7)	0.451
Interleukin 6 (pg/mL)^d^	10.2 (4.2–20.4)	12.4 (3.5–28.5)	0.510
Ferritin (ng/mL)	232 (99–446)	462 (276–885)	**0.002**
C Reactive Protein (mg/L)	3.1 (0.5–8.8)	7.4 (3.8–13.8)	** < 0.001**
**Biochemical markers**			
Glucose (mg/dL)	105.5 (83–115.2)	108 (90–142.5)	0.098
Cholesterol (mg/dL)	138.5 (108.2–179.2)	144 (122–166)	0.965
Urea (mg/dL)	32 (27–44)	38 (30–51)	0.079
Creatinine (mg/dL)	0.8 (0.7–1)	0.8 (0.7–1)	0.704
Aspartate aminotransferase (AST) (U/L)	29 (23–41)	34(25–46)	0.129
Alanine aminotransferase (ALT) (U/L)	29 (17–44)	33 (22–59)	0.121
Albumin (g/dL)	3.7 (3.3–4.2)	4 (3.6–4.2)	0.090
Lactate dehydrogenase (LDH) (U/L)	236 (187–296)	285 (238–348)	** < 0.001**
Troponin (ng/L)^e^	3 (0–5)	6 (2–14)	**0.010**

Data are presented as n (%) or median (25th-75th interquartile range)

^a^The No Pneumonia group refers to the ambulatory group comprises no without presenting pneumonia—WHO 1

^b^The Pneumonia group comprises mild illness with pneumonia—WHO 2–7

^c^No Pneumonia and Pneumonia groups were compared using the non-parametric Mann–Whitney test for continuous data and √χ^2^ test for categorical data. P-value < 0.05 was considered significant and marked in bold

^d^IL-6 data were from 244 patients, WHO 1 n = 18 and WHO 2–7 n = 226

^e^Troponin data were from 133 patients, WHO 1 n = 25 and WHO 2–7 n = 108

**Table 2:** Baseline comorbidities of the COVID-19 study cohort and COVID-19 infection treatment used classified by pneumonia

Variables	No Pneumonia^a^ (WHO 1) (n = 24)	Pneumonia^b^ (WHO 2–7) (n = 228)	P-value^c^
**Comorbidities—no. (%)**			
Obesity	10 (23.8)	123 (38)	0.528
Diabetes Mellitus (DM)	7 (16.7)	67 (20.7)	0.353
Hypertension (HTA)	15 (35.7)	148 (45.7)	0.145
Dyslipidemia (DLP)	10 (23.8)	113 (34.9)	0.103
Cardiovascular diseases (CVD)	7 (16.7)	38 (11.7)	0.244
Respiratory diseases	5 (11.9)	32 (9.9)	0.426
Cancer	6 (14.3)	35 (10.8)	0.324
**COVID-19 treatment**			
Oxygen requirement	0 (0)	28 (12.3)	**0.042**
Hydroxychloroquine	0 (0)	22 (9.6)	0.104
Azithromycin	3 (12.5)	142 (62.2)	**< 0.001**
Kaletra	0 (0)	16 (7)	0.171
Tocilizumab	0(0)	12 (5.3)	0.093
Interferon	0(0)	1 (0.4)	0.740
Corticosteroids	6 (20.8)	177 (77.6)	**< 0.001**
Remdesivir	1 (4.2)	58 (25.4)	**0.016**

Data are presented as n (%)

^a^The No Pneumonia group refers to the ambulatory group comprises no without presenting pneumonia –WHO 1

^b^The Pneumonia group comprises mild illness with pneumonia -WHO 2–7

^c^No Pneumonia and Pneumonia groups were compared using the non-parametric Mann–Whitney test for continuous data and √χ^2^ test for categorical data. P-value < 0.05 was considered significant and marked in bold

The Ig concentrations are independent of the presence of previous comorbidities (Fig. 2C). IgA concentrations were positively correlated with age in both men (r = 0.143; p = 0.04) (Fig. 2D) and women (r = 0.196; p = 0.042) who developed COVID-19 pneumonia (Fig. 2E).

### IgA is strongly associated with COVID-19 pneumonia development

ROC (receiver operating characteristic) curves were performed for each Ig to evaluate their discriminatory ability to correctly assign patients into COVID-19 pneumonia group at the acute phase of SARS-CoV-2 infection.

**Fig. 3:**
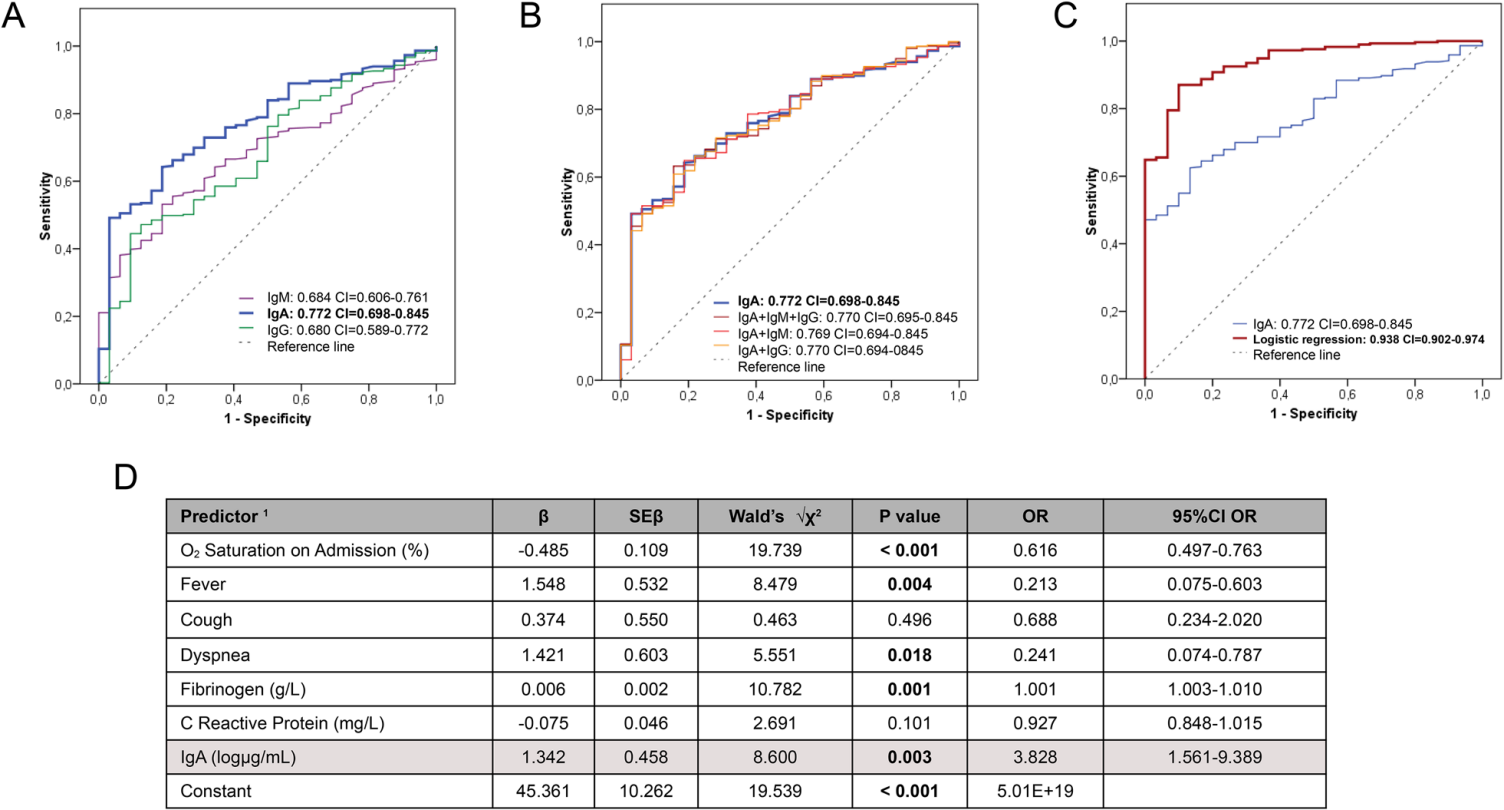
Biomarker analysis to discriminate SARS-CoV-2 positives developing COVID-19 pneumonia from asymptomatic. **A** Receiver operating characteristic (ROC) curves comparing the No Pneumonia group (WHO 1) to Pneumonia (WHO 2–7) group for serum IgM, IgA and IgG concentrations and **B** the different combinations of IgA with the others Igs. **C** ROC curve of serum IgA levels together with other previously related factors to COVID-19 severity by binary logistic regression. All ROC curves were performed using IBM SPSS Statistics 21.0. **D** Information of logistic regression analysis of combined selected factors, including circulating IgA concentrations, to differentiate between subjects developing COVID-19 pneumonia and those without developing COVID-19 pneumonia in the COVID-19 study cohort at the time of admission. Predictor variables were coded as follows: fever no = 0, fever yes = 1; cough no = 0, cough yes = 1; dyspnea no = 0, dyspnea yes = 1. P-value < 0.05 was considered significant and marked in bold

IgA was the immunoglobulin with the highest discriminative power for COVID-19 pneumonia according to the area under the ROC curve (AUC) values (0.772 (CI 0.698–0.845)) and with a sensitivity of 65% and a specificity of 78% (Fig. 3A). Models combining IgA with IgM and/or IgG concentrations corroborated that IgA is the best candidate to correctly assign SARS-CoV-2 infected patients into the ambulatory and COVID-19 pneumonia groups during the acute phase (Fig. 3B). A logistic regression was also performed to ascertain the discriminatory power of combining circulating IgA with selected factors related to disease severity, such as fever, cough, dyspnea, fibrinogen (g/L) and CRP (mg/L). The likelihood that participants will have COVID-19 pneumonia obtained an AUC of 0.938 (CI 0.938–0.974) (Fig. 3C, D). The logistic regression model was statistically significant, χ^2^(7) = 5.687, P < 0.001. The model explained 54.2% (Nagelkerke R2) of the variance in COVID-19 pneumonia and correctly classified 93.2% of cases. SARS-CoV-2-infected patients with high circulating IgA concentrations in the acute phase of the infection were 3.828 times more likely to exhibit COVID-19 pneumonia (Fig. 3C).

### IgM concentration in COVID-19 pneumonia depended on previous comorbidities

**Fig. 4:**
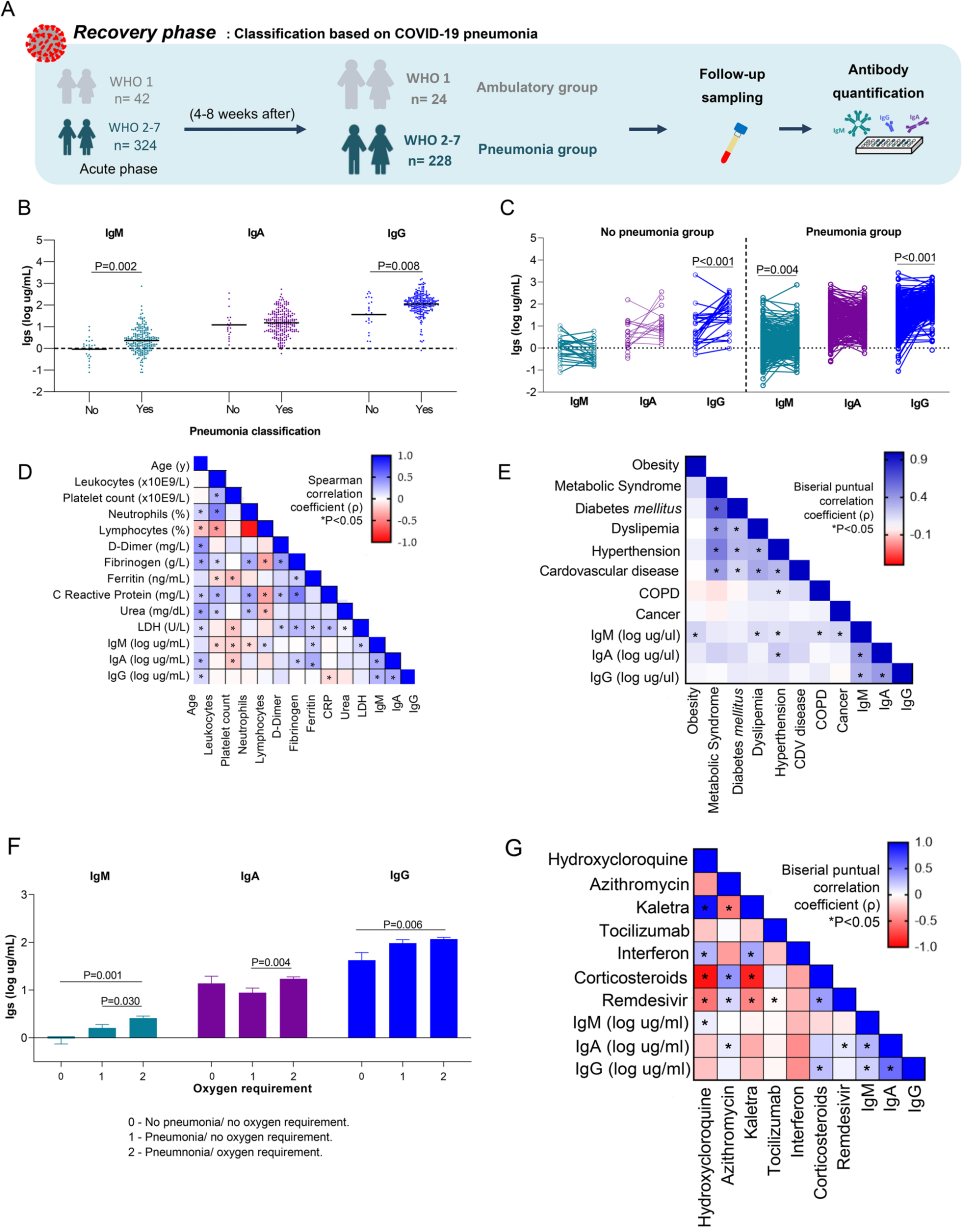
Circulating levels of immunoglobulins at 4–8 weeks of infection based on pneumonia development. **A** The longitudinal cohort study included 252 of these patients who were followed up after 4 to 8 weeks (recovery phase). Blood sampling for ELISA analysis was performed after 4 to 8 weeks (recovery phase). **B** Circulating levels of IgM, IgA, and IgG at 4–8 weeks of infection in the No Pneumonia (WHO 1; patients without pneumonia) and Pneumonia group (WHO 2–7). Scatter dot plot with mean (wide line). Statistical differences among groups were determined by the nonparametric Mann–Whitney U-test. **C** Differences in IgM, IgA, and IgG concentrations at 4–8 weeks compared to the acute phase. Statistical differences among time points were determined by the Wilcoxon test. **C** Heatmap showing the Spearman (ρ) correlation coefficient between serum IgM, IgA and IgG concentrations and age and selected biochemical parameters at recovery phase in pneumonia group (WHO 2–7) based on pneumonia development. **D** Heatmap showing the point-biserial correlation coefficient (pb) of pairwise comparison analyses between previous comorbidities with levels of the three Igs at 4–8 weeks. Spearman (ρ) matrix and point-biserial (pb) matrix are colour-coded (− 1:1, red: blue through white), and correlations with statistical significance are indicated with an asterisk as *P < 0.05. **E** Relationship between oxygen requirements and Igs concentrations. Group 0 corresponded to WHO 1 patients without pneumonia, Group 1 corresponded to WHO 2–3 patients with pneumonia but not oxygen requiring and Group 2 corresponded to WHO 4–7 patients with pneumonia and not oxygen requiring. **F** Heatmap showing the point-biserial correlation coefficient (pb) of pairwise comparison analyses between COVID-19 treatment administrated during acute phase with levels of the three Igs at 4–8 weeks in pneumonia group. Point-biserial (pb) matrix is colour-coded (− 1:1, red: blue through white), and correlations with statistical significance are indicated with an asterisk as *P < 0.05

Circulating Ig concentrations were analysed in 252 patients with follow-up between 4 and 8 weeks after the infection (recovery phase): 24 patients in the ambulatory (WHO1, no pneumonia) and 228 patients in the COVID-19 pneumonia group (WHO2-7) (Fig. 4A). The recovery phase cohort was composed of 50% males with an average age of 54 years in the ambulatory group and 61.8% males with an average age of 59 in the COVID-19 pneumonia group (Table 3). There were no differences in hematological, inflammatory, or biochemical markers between groups except for fibrinogen, which was significantly higher in COVID-19 pneumonia patients (P < 0.001) (Table 3).

**Table 3:** Clinical and biochemical characteristics of the COVID-19 study cohort in the recovery phase of infection

Variables	No Pneumonia^a^ (WHO 1) (n = 24)	Pneumonia^b^ (WHO 2–7) (n = 228)	P-value^c^
Male	12 (50)	141 (61.8)	0.259
Age, years	54 (40.7–62.5)	59 (50–68)	**0.025**
**Hematological parameters**			
Leukocytes (×10E9/L)	5.9 (4.8–6.5)	5.7 (4.7–7.3)	0.882
Red blood cell count (×10E9/L)	4.6 (4–5.1)	4.5 (4.2–4.8)	0.395
Hemoglobin (g/dL)	13.7 (12.2–14.8)	13.2 (12.2–14.3)	0.284
Hematocrit (%)	42.5 (38.1–46)	41.3 (37.8–44)	0.218
Platelet count (×10E9/L)	237.5 (184.2–284.2)	228.5 (189–283)	0.892
Neutrophils (%)	56.2 (50–59)	54.5 (47.8–61.1)	0.896
Lymphocytes (%)	33.7 (27.5–39.3)	31.5 (26–37.8)	0.309
Total lymphocytes	1855 (1655–2517.5)	1815 (1440–2292.5)	0.193
Monocytes (%)	8.3 (7.2–9.7)	8.8 (7.4–10.2)	0.411
**Inflammatory markers**			
d-Dimer (mg/L)	418.5 (297–696.7)	426 (331–686)	0.459
Fibrinogen (g/L)	426 (352.7–462.5)	505.5 (426.7–592)	** < 0.001**
Interleukin 6 (pg/mL)^d^	2.7 (0.5–2.8)	2.2 (0.6–4)	0.770
Ferritin (ng/mL)	129 (68–292.5)	180 (94–340)	0.185
C Reactive Protein (mg/L)	0.2 (0–0.4)	0 (0–0.7)	0.831
**Biochemical markers**			
Glucose (mg/dL)	93.5 (85.5–107.5)	98.5 (89–114)	0.250
Urea (mg/dL)	36 (27.7–47)	36 (29–44)	0.711
Creatinine (mg/dL)	0.87 (0.8–0.9)	0.8 (0.7–1)	0.474
Aspartate aminotransferase (AST) (U/L)	22 (19–27.2)	23 (18–28)	0.782
Alanine aminotransferase (ALT) (U/L)	25 (19.7–34.2)	30 (21–46)	0.181
Lactate dehydrogenase (LDH) (U/L)	193 (170–210)	197 (175–221)	0.333

Data are presented as n (%) or median (25th–75th interquartile range)

^a^The No Pneumonia group refers to the ambulatory group comprising mild illness without pneumonia—WHO 1

^b^The Pneumonia group comprises mild illness with pneumonia—WHO 2–7

^c^No Pneumonia and Pneumonia groups were compared using the non-parametric Mann–Whitney test for continuous data and √χ^2^ test for categorical data. P-value < 0.05 was considered significant and marked in bold

^d^IL-6 data were from 244 patients, WHO 1 n = 21 and WHO 2–7 n = 188

At the recovery phase, IgM and IgG concentrations were higher in the COVID-19 pneumonia group than in the ambulatory group (P = 0.001 and P = 0.003, respectively), but no significant difference in IgA concentration was observed between groups (Fig. 4B). IgG concentrations were higher over time in both groups (P < 0.001). IgM concentrations were higher in the recovery phase compared to the acute phase in the COVID-19 pneumonia group (P = 0.004). No significant differences in IgA concentrations were observed between the two time points in either group (Fig. 4C).

Correlation analyses were also performed to better understand the Ig response to COVID-19 pneumonia. IgM was positively correlated with lymphocytes (ρ = 0.140, P = 0.043), ferritin (ρ = 0.182, P = 0.013), and LDH (ρ = 0.169, P = 0.019). IgA was positively correlated with fibrinogen (ρ = 0.198, P = 0.005) and ferritin (ρ = 0.324, P < 0.001). IgA and IgG were positively related to age (ρ = 0.250, P < 0.001 for IgA and ρ = 0.151, P = 0.023 for IgG). IgM was negatively correlated with leukocytes (ρ = − 0.153, P = 0.029), platelets (ρ = − 0.193, P = 0.006), and neutrophils (ρ = − 0.149, P = 0.033). IgA was negatively related to platelets (ρ = − 0.224, P = 0.001), IgG was negatively correlated with CRP (ρ = − 0.154, P = 0.029) (Fig. 4D). In contrast to what happens in the acute phase, the presence of previous comorbidities correlated with different Ig concentrations. IgM levels were positively correlated with obesity (ρb = 0.156, P = 0.020), dyslipemia (ρb = 0.140, P = 0.029), COPD (ρb = 0.133, P = 0.037) and cancer (ρb = 0.173, P = 0.007). Hypertension was significantly correlated with both IgM (ρb = 0.148, P = 0.020) and IgA (ρb = 0.178, P < 0.001) (Fig. 4E and Additional file 1: Fig. S1).

### Ig concentrations in COVID-19 pneumonia were affected by COVID-19 treatments

Ig concentrations at the recovery phase were evaluated according to respiratory and hemodynamic measures indicated in the COVID-19 treatment. IgM concentrations were significantly higher in COVID-19 pneumonia patients who required oxygen therapy (group 2) compared to both patients who developed pneumonia but did not require oxygen therapy (group 1, P = 0.030) and patients who did not develop COVID-19 pneumonia (group 0, P = 0.001). IgG concentrations were significantly higher in COVID-19 pneumonia patients who required oxygen therapy (group 2) compared to patients who did not develop COVID-19 pneumonia (group 0, P = 0.006). IgA concentrations were significantly higher in the pneumonia group with an oxygen requirement compared with patients who developed pneumonia but did not require oxygen therapy (group 1, P = 0.004) (Fig. 4F).

In relation to COVID-19 drug in pneumonia group, hydroxychloroquine was positive correlated with IgM concentrations (ρb = 0.132, P = 0.050). Corticosteroids were significant correlated with IgG concentrations (ρb = 0.181, P = 006). Remdesivir and Azithromycin were related with IgA concentrations (ρb = 0.140, P = 0.037 and ρb = 0.133, P = 0.048 respectively) (Fig. 4G and Additional file 1: Fig. S2).

## Discussion

Respiratory failure and COVID-19 pneumonia development are the most common complications of SARS-CoV-2 infection, which represents a serious clinical issue [20, 21]. Immunoglobulins are part of the adaptive immune system; they can reflect the state of disease through great variation in circulating concentrations over time [23]. In the present study, SARS-CoV-2-infected patients were classified into two groups based on the presence or absence of COVID-19-related pneumonia. Circulating IgM, IgA and IgG concentrations were analyzed at the acute phase (time of admission, baseline) in unvaccinated 320 well-characterized patients with a positive diagnosis of SARS-CoV-2 infection and in 252 patients with follow-up between 4 and 8 weeks after the time of admission (recovery phase). The correlation between IG concentrations and COVID-19 treatment was also assessed to enhance the reliability of our findings. The study of SARS-CoV-2 unvaccinated patients offers a better interpretation and comprehension of the humoral immune response in the SARS-CoV-2 infection and the development of COVID-19 pneumonia than including a combination of patients with different-dose vaccine stages due to the different cellular responses could be observed in vaccinated people [24, 25].

The present work, in accordance with previous studies [26, 27], showed that at the acute phase, IgA concentrations discriminate patients who will develop COVID-19 pneumonia from those who will not, independently of the presence of previous comorbidities, although age is a factor that was positively correlated with the levels of this immunoglobulin. High IgA concentrations together with the most common non-respiratory and respiratory defining symptoms of COVID-19 severity (fever, dyspnea, cough, O2 saturation on admission, CRP and fibrinogen), obtained a predictive model with 93% reliability to detect the risk of COVID-19 pneumonia development. Previous studies identified IgA levels at the beginning of SARS-CoV-2 infection as an independent predictor of disease severity in patients with COVID-19 [12, 28, 29] and the main isotope produced as a consequence of SARS-CoV-2 infection [30]. IgA is the second most abundant antibody isotype in human serum and its function related to infection and inflammatory homeostasis in mucosal surfaces has been extensively characterized; in innate immune cells, IgA is involved in both pro-inflammatory (the myeloid-cell-specific type I Fc receptor (FcαRI)-dependent responses induced by IgA immune complexes) and anti-inflammatory pathways (FcαRI upon binding of monomeric IgA) [31]. In human serum exists two subtypes of IgA isotope, IgA1 and IgA2, which are distributed proportionally about 90% and 10%, respectively [32]. Surprisingly, the role in human serum has been poorly understood compared to that of the mucosa and the serum IgA function in COVID-19 severity is still controversial [31]. In this regard, our findings support the hypothesis that an early IgA response is indicative of potential negative effects on COVID-19 progression [33]. High production of IgA autoantibodies, specially elevated IgA2 subclass, corresponds with a pathogenic and inflammatory role of IgA in multiple inflammatory diseases [34, 35]. Serum IgA concentrations has been positively correlated with high levels of inflammation, which denotes that IgA has a key role in the disturbance of the cytokine network and it is decisive in shaping immune responses [26, 31]. Of note, IgA2 subclass is a potential inductor of neutrophil extracellular trap (NET) formation and the first effector of proinflammatory component of IgA responses [36]. Indeed, our results corroborated a strong association between serum IgA and inflammatory markers such as erythrocyte sedimentation rate (ESR) or ferritin. The increase of serum IgA levels in COVID-19 pneumonia could be indicative of an over-activation of the immune system. Prolonged overstimulation over time induces hyperinflammation which could ends up causing damage to tissues and organs [34, 35], as in, for example, the acute respiratory distress syndrome (ARDS) where damage occurs in the respiratory system [5]. Our findings, although they do not correspond to the current global COVID-19 situation, corroborate the importance of maintaining an early healthy balance between pro-inflammatory and anti-inflammatory mechanisms in SARS-CoV-2 infection to prevent these clinical complications or when developing IgA monoclonal antibodies (mAbs) as a therapeutic option [37].

At the recovery phase, IgM and IgG concentrations were found higher in patients who developed COVID-19 pneumonia. These results were consistent with the previously described peak titter for IgM or IgG at 15–35 days post-symptom onset of SARS-CoV-2 infection [33, 38–40]. The role and kinetics of secreted IgM in disease progression are poorly studied, and this is partly because IgM has commonly been identified as a feature of primary infection by pathogens rapidly followed by conversion to IgG. However, the IgM response could persist beyond disease progression, as our results have shown in agreement with the previously described response to multiple infectious diseases. The circulation of this immunoglobulin over time could correlate with a worse disease progression [23, 41, 42]. Significant increase in circulating IgM levels in the pneumonia group was positively correlated with markers of inflammation. In the case of chronic or extreme inflammation, IgM can promote inflammation and tissue damage due to its access to tissues that are otherwise inaccessible. Of note, the presence of comorbidities such as obesity, hypertension, respiratory or oncological diseases affected IgM production increasing their concentrations in COVID-19 pneumonia [43–47]. Our results are consistent with previous studies suggesting that pre-existing conditions may predispose patients to an unfavorable clinical course and an increased risk of intubation and death [44–46, 48]. Otherwise, IgM production is usually followed by high-affinity IgG, which is crucial for long-term immunity or immunological memory after infection [49]. Our results corroborated that all positive SARS-CoV-2 patients had a significant increase in circulating IgG concentration from the acute phase to the recovery phase [50]. It is also important to highlight that the IgG levels, in the COVID-19 pneumonia group, were associated with the use of corticosteroids, the most common drugs employed to suppress inflammation produced by COVID-19 infection [51]. The use of corticosteroids is controversial; they could have an effect on the production of the Igs in COVID-19 pneumonia. Although it has been described that short-term corticosteroid therapy does not influence IgG kinetics, its use has been implemented in COVID-19 severe patients to treat inflammation. Of note, corticosteroid treatment in *Pneumocystis pneumonia* induces down-regulation of genes related to B-cell signalling, homeostasis and Ig production [52, 53]. The effects of corticosteroids on cellular function are poorly studied, and further studies would be required to determine the molecular pathways that are affected.

### Limitations of study

The present study included a low level of variability in the age range. To solve this problem, more patients in younger age ranges in both groups will be required to assess whether age is a key factor that influences immunoglobulin levels in COVID-19 pneumonia development. The study only included people from March 2020 to February 2021, the Spanish first to third waves, which is far from the current situation with this pandemic. The vaccine mostly impacts the humoral response, inducing different cellular responses; a well-matched, characterized cohort of unvaccinated SARS-CoV-2 patients provides a better comprehension of the humoral response (the objective of our study) in the severity of COVID-19 pneumonia. On the other hand, the limited participation of re-infected patients with different virus variants facilitates the interpretation of our results. Igs measurements have performed using the NP antigen; further experiments using different proteins or pools of proteins of SARS-CoV-2 may be useful to validate our results. Finally, in the future, it would be interesting to determine whether secretory antibodies may contribute to long-term barrier tissue damage in COVID-19 pneumonia and evaluate the correlation between circulating immunoglobulin concentrations and their presence in different tissues.

## Conclusions

IgA has been strongly associated with increased risk of COVID-19 pneumonia development whereas circulating IgG will be crucial in the diagnosis of active COVID-19 and could be related to long-term immunity in all COVID-19 positives. During the recovery phase, high levels of immune/induced IgM will be associated to COVID-19 pneumonia and could be involved in tissue damage [12]. Also, IgM concentrations have been correlated with previous comorbidities or COVID-19 therapy used [12]. In this regard, there are not many studies that focus on the study of the influence of the treatments used in COVID-19 pneumonia on the levels of the different immunoglobulins in the long term. Our data confirmed that the interaction of different pharmacological interventions and the presence of respiratory complications needs to be explored. Overall, our results could contribute to a better understanding of the humoral response at each stage of disease development and help in the development of more effective treatments and vaccines.

### Supplementary Information

**Additional file 1: Figure S1.** Comorbidities and immunoglobulin concentrations during the recovery phase. Levels of the different immunoglobulins based on the comorbidities and pneumonia of the significative correlations resulting from the point-biserial matrix. No or yes indicates absence or presence of pneumonia and the numbers in parenthesis indicate n of the group. Statistical differences among groups were determined by the nonparametric Kruskal–Wallis test followed by Mann–Whitney U-test. Abbreviations: CVD: cardiovascular diseases, COPD: Respiratory diseases and HTA: hypertension. **Figure S2.** Pneumonia treatments and immunoglobulins during the recovery phase. Levels of the different Igs based on the treatments and pneumonia of the significative correlations resulting from the point-biserial matrix. No or yes indicates absence or presence of pneumonia and the numbers in parenthesis indicate n of the group. Statistical differences among groups were determined by the nonparametric Kruskal–Wallis test followed by Mann–Whitney U-test. Abbreviations: HCQ: Hydroxycloroquine and CORTICOIDS: Corticostereroids.

**Additional file 2: Table S1.** Heamatological and biochemical parameters of the COVID-19 study cohort at baseline. Data are presented as n (%) or median (25th–75th interquartile range).

## Data Availability

The data that support the findings of this study are available from the corresponding author, A Rull (anna.rull@iispv.cat), upon reasonable request.

## Abbreviations

AST: Aspartate aminotransferase
ALT: Alanine aminotransferase
ARDS: Acute respiratory syndrome
AUC: Area under curve
CRP: C reactive protein
CVD: Cardiovascular diseases
DM: Diabetes Mellitus
DLP: Dyslipidemia
ELISA: Enzyme-linked immunosorbent assay
ESR: Erythrocyte sedimentation rate
FcαRI: Myeloid-cell-specific type I Fc receptor
HTA: Hypertension
ICU: Intensive Care Unit
Ig: Immunoglobulin
IgA: Immunoglobulin A
IgG: Immunoglobulin G
IgM: Immunoglobulin M
LDH: Lactate dehydrogenase
mAbs: Monoclonal antibodies
PTE: Pulmonary thromboembolism
ROC: Receiver operating characteristic
SARS-CoV-2: Severe acute respiratory syndrome coronavirus-2
SD: Standard deviation
SEM: Standard error of the mean
