# Supplementary figures and images for: Immunoglobulins in COVID-19 pneumonia: from the acute phase to the recovery phase
Source: Eur J Med Res. 2024 Apr 6;29:223. doi: 10.1186/s40001-024-01824-5 (PMC10998353; doi:10.1186/s40001-024-01824-5)

Figure S1

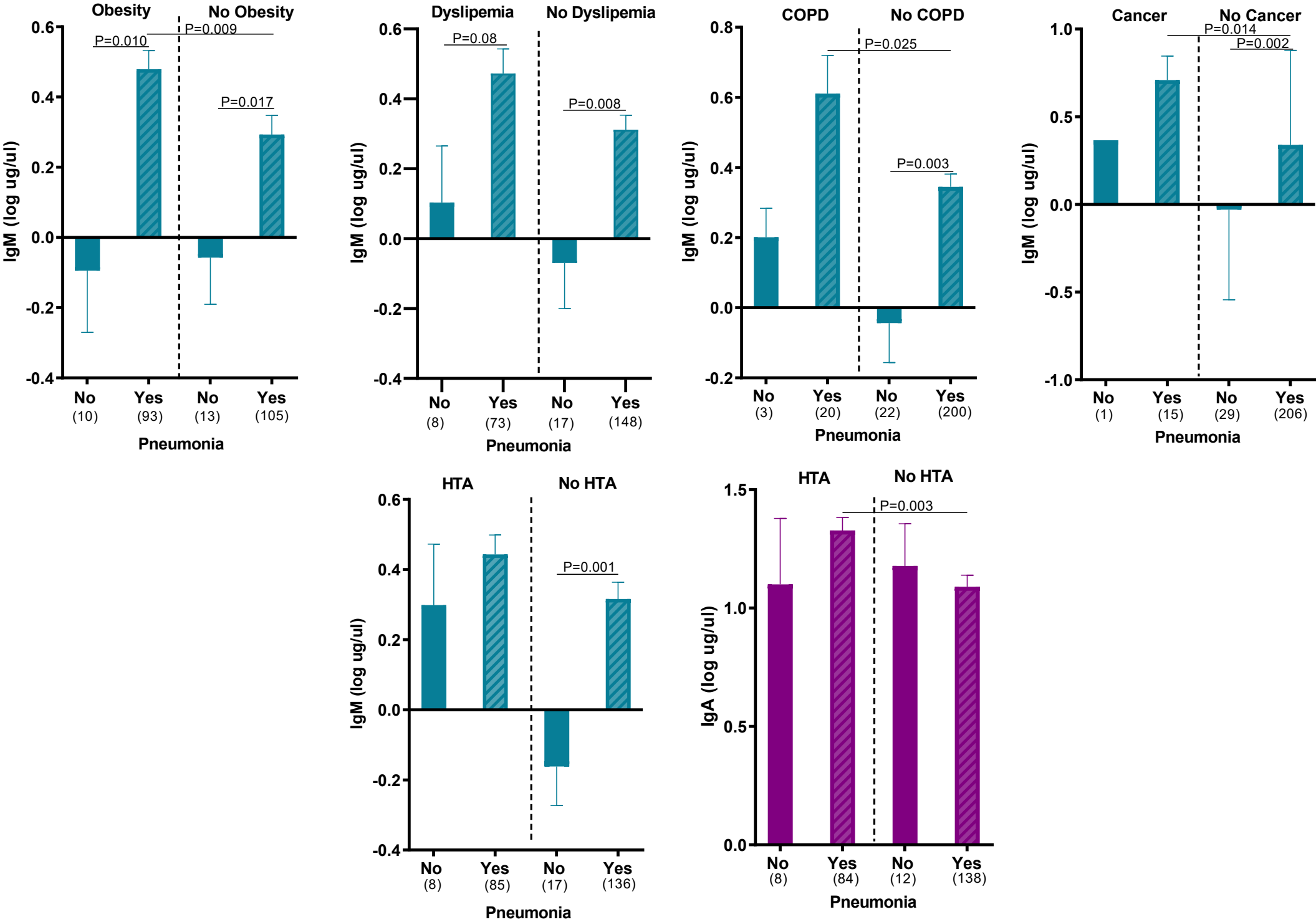

Figure S2

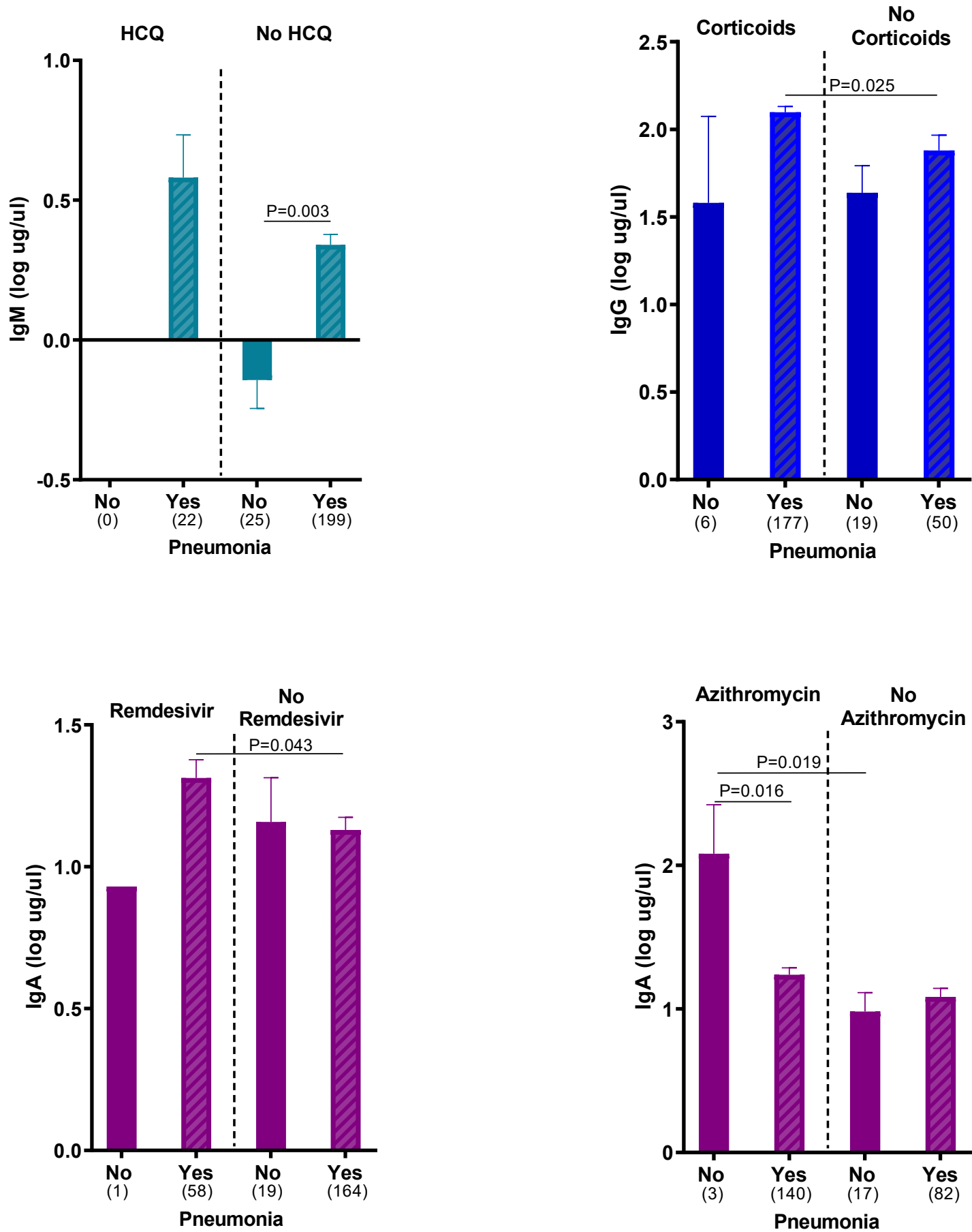

Supplement: Supplementary file 1 — Additional file 1: Figure S1. Comorbidities and immunoglobulin concentrations during the recovery phase. Levels of the different immunoglobulins based on the comorbidities and pneumonia of the significative correlations resulting from the point-biserial matrix. No or yes indicates absence or presence of pneumonia and the numbers in parenthesis indicate n of the group. Statistical differences among groups were determined by the nonparametric Kruskal–Wallis test followed by Mann–Whitney U-test. Abbreviations: CVD: cardiovascular diseases, COPD: Respiratory diseases and HTA: hypertension. Figure S2. Pneumonia treatments and immunoglobulins during the recovery phase. Levels of the different Igs based on the treatments and pneumonia of the significative correlations resulting from the point-biserial matrix. No or yes indicates absence or presence of pneumonia and the numbers in parenthesis indicate n of the group. Statistical differences among groups were determined by the nonparametric Kruskal–Wallis test followed by Mann–Whitney U-test. Abbreviations: HCQ: Hydroxycloroquine and CORTICOIDS: Corticostereroids. [file 40001_2024_1824_MOESM1_ESM.pdf]
