# Supplementary material for: Immunoglobulins in COVID-19 pneumonia: from the acute phase to the recovery phase
Source: Eur J Med Res. 2024 Apr 6;29:223. doi: 10.1186/s40001-024-01824-5 (PMC10998353; doi:10.1186/s40001-024-01824-5)
Supplement: Supplementary file 2 — Additional file 2: Table S1. Heamatological and biochemical parameters of the COVID-19 study cohort at baseline. Data are presented as n (%) or median (25th–75th interquartile range). [file 40001_2024_1824_MOESM2_ESM.docx]

**Table S1.** Heamatological and biochemical parameters of the COVID-19 study cohort at baseline. Data are presented as n (%) or median (25^th^-75^th^ interquartile range).

|  | **No pneumonia^1^** |  | **Pneumonia^2^** | | |  |
| --- | --- | --- | --- | --- | --- | --- |
|  | **Ambulatory**  **(WHO 1)**  (n=42) |  | **Mild**  **(WHO 2-3)**  (n=47) | **Severe**  **(WHO 4-5)**  (n=182) | **Critical**  **(WHO 6-7)**  (n=49) | **P-value^3^** |
| **Haematological Parameters** | | | | | |  |
| Leukocytes (x10E9/L) | 6.1 (4.8-8.1) |  | 5.3 (4-6.9) | 6.4 (4.5-8.2) | 7.4 (5.1-9.9) | **0.003** |
| Red blood cell count (x10E9/L) | 4.3 (3.7-4.9) |  | 4.5 (4.2-4.9) | 4.5 (4-4.9) | 4.5 (4.1-5) | **0.028** |
| Hemoglobin (g/dL) | 12.8 (10.4-14.7) |  | 13 (12.5-13.8) | 13(11.9-14) | 13.2 (11.7-14.4) | 0.058 |
| Hematocrit (%) | 38 (31.8-44.8) |  | 40 (38.2-42.6) | 39 (36.8-42.8) | 30.3 (36.5-43.7) | 0.132 |
| Platelet count (x10E9/L) | 206  (172.5-235) |  | 223  (188-264) | 229  (168-295) | 225.5  (166-310.7) | **0.023** |
| Neutrophils (%) | 68.8  (58.7-79.7) |  | 63.2  (51.3-73.9) | 75.7  (66.7-82.6) | 80.7  (72.3-88.1) | **<0.001** |
| Lymphocytes (%) | 21.5  (12-28.8) |  | 24.4  (18.7-37.2) | 17.7  (10.8-24.7) | 12.2  (7.9-20.9) | **<0.001** |
| Total lymphocytes (%) | 1215  (755-1592.5) |  | 1501.4  (980-1900) | 990  (740-1370) | 760  (582.5-1310) | **<0.001** |
| Monocytes (%) | 8.0 (5.8-10.4) |  | 8.4 (6.6-10.2) | 6.2 (4.9-8.4) | 5.5 (4.2-7.8) | **0.007** |
| **Coagulation Parameters** | |  |  |  |  |  |
| Activated thromboplastin time (seg) | 29.5 (26.5-32.1) |  | 30.4 (29-32.8) | 31 (28.5-33.1) | 30.4 (29-33) | 0.283 |
| Prothrombin time (seg) | 12.9 (11.7-13.4) |  | 12.7 (12.4-13.2) | 12.7 (11.9-13.4) | 13.4 (12.2-14.3) | 0.144 |
| D-Dimer (mg/L) | 501.5  (316.2-1191.5) |  | 600  (378-932) | 566.5  (399.7-821.2) | 833.5  (596.7-1371.5) | **<0.001** |
| Fibrinogen (g/L) | 561 (435-761 ) |  | 687 (598-784) | 780 (681-869) | 783 (685.7-893.5) | **<0.001** |
| **Inflammatory Markers** | | | | | |  |
| Erythrocyte sedimentation rate (mm) | 89  (14-117.5) |  | 51.5  (21.7-79.2) | 52.5  (32-83.2) | 52  (31-81.5) | 0.399 |
| Interleukin 6 (pg/ml)^4^ | 10.2 (4.2-20.4) |  | 4.8 (1.8-14.8) | 11.7 (3.4-27.9) | 22.9 (7.7-39.6) | **<0.001** |
| Ferritin (ng/ml) | 232  (99-446) |  | 407  (154-598.5) | 450  (260-870.7) | 798  (413-1375) | **<0.001** |
| C Reactive Protein (mg/L) | 3.1 (0.5-8.8) |  | 3.6 (1.8-6.5) | 7.9 (4.2-12.2) | 9.1 (4.4-16.6) | **<0.001** |
| **Biochemical Markers** | | | | | |  |
| Glucose (mg/dL) | 105.5  (83-115.2) |  | 93  (84-114) | 104  (87.7-136) | 126  (105.5-195.5) | **<0.001** |
| Cholesterol (mg/dL) | 138.5  (108.2-179.2) |  | 156  (133-179) | 144  (121.7-163) | 127.5  (122.2-149.2) | **0.037** |
| Urea (mg/dL) | 32 (27-44) |  | 33 (27-40) | 35 (27-44.2) | 45 (35-66) | **<0.001** |
| Creatinine (mg/dL) | 0.81 (0.7-1) |  | 0.8 (0.6-0.9) | 0.8 (0.6-0.9) | 0.9 (0.7-1.1) | **<0.001** |
| Aspartate aminotransferase  (AST) (U/L) | 29 (23-41) |  | 30 (25-41) | 34 (24-46.2) | 37 (29.2-58) | 0.181 |
| Alanine aminotransferase (ALT) (U/L) | 29 (17-44) |  | 32 (23-51) | 33 (22-60.2) | 37 (24.7-69.7) | **0.025** |
| Albumin (g/dL) | 3.7 (3.3-4.2) |  | 4.2 (4-4.4) | 3.9 (3.7-4.2) | 3.8 (3.5-4) | **<0.001** |
| Lactate dehydrogenase  (LDH) (U/L) | 236  (187-296) |  | 245.5  (210.5-292.5) | 282  (239.7-343.5) | 342  (275-435) | **<0.001** |
| Troponin (ng/L)^5^ | 3 (0-5) |  | 2 (0-4) | 5 (3-13) | 6.5 (2.2-9.5) | **<0.001** |

^1^ The ambulatory group comprises mild illness without pneumonia –WHO 1.

^2^ The mild group comprises mild illness with pneumonia without hospitalization-WHO 2 or with hospitalization-WHO 3, but not oxygen requiring. The severe group comprises moderate pneumonia requiring low-flow oxygen-WHO 4 and/or non-invasive ventilation-WHO 5. The critical group comprises severe pneumonia requiring mechanical ventilation/intubation-WHO 6 and vasopressors or dialysis-WHO 7.

^3^ No Pneumonia and Pneumonia groups were compared using the non-parametric Mann-Whitney test for continuous data and √ꭕ2 test for categorical data. P-value < 0.05 was considered significant and marked in bold.

^4^ IL-6 data were from 244 patients, WHO 1 n=18, WHO 2-3 n=38, WHO 4-5 n=160 and WHO 6-7 n=28.

^5^ Troponin data were from 133 patients, WHO 1 n=25, WHO 2-3 n=24, WHO 4-5 n=74 and WHO 6-7 n=10.
